# Detection, fate and transport of the biohazardous agent *Toxoplasma gondii* in soil water systems: Influence of soil physicochemical properties, water chemistry and surfactant

**DOI:** 10.1111/1758-2229.13204

**Published:** 2023-09-22

**Authors:** Erin N. Kinsey, Caroline Korte, Sohib Gouasmia, Coralie L'Ollivier, Jitender P. Dubey, Aurélien Dumètre, Christophe J.G. Darnault

**Affiliations:** ^1^ Department of Environmental Engineering and Earth Sciences, School of Civil and Environmental Engineering and Earth Sciences Clemson University Anderson South Carolina USA; ^2^ Aix Marseille University Marseille France; ^3^ IHU‐Méditerranée Infection Marseille France; ^4^ AP‐HM, Parasitology Laboratory Timone Hospital Marseille France; ^5^ United States Department of Agriculture, Agricultural Research Service, Animal Parasitic Diseases Laboratory Beltsville Agricultural Research Center Beltsville Maryland USA

## Abstract

A series of laboratory experiments were conducted to study the fate and transport of *Toxoplasma gondii* oocysts in soils as a function of soil physicochemical properties and soil water chemistry properties. Soil columns were homogeneously packed with loamy sand soils (Lewiston and Greenson series) and sandy loam soils (Sparta and Gilford series), and subject to hydrologic conditions characterized by the absence and presence of an anionic surfactant—Aerosol 22 in the artificial rainfall. Quantitative polymerase chain reaction (qPCR) was utilized for the detection and enumeration of oocysts in soil leachates to evaluate their breakthrough and in soil matrices to examine their spatial distribution. Differences in the rate and extent of transport of oocysts were observed as a function of physical and chemical parameters tested. The breakthrough of oocysts was observed for all the soils irrespective of the presence of surfactant. However, in the absence of surfactant, the predominant fate of oocysts in soils subject to simulated rainfall was their retention in the soil profile. The presence of surfactant induced a change in the fate of oocysts in these soils exposed to rainfall simulation as the predominant fate of oocysts was found to be in the soil leachates.

## Graphical abstract

Laboratory set‐up for flow‐through experiments of soil columns under rainfall simulator in a biosafety cabinet; Photomicrograph of *Toxoplasma gondii* oocysts; Photos of soil column and soil column section; Breakthrough curves of oocysts in soils (oocysts (C/C_o_) vs. pore volume, where C_o_ is the concentration of oocysts in the inoculums applied to the surface of the soil columns, and C refers to the concentration of oocysts in leachates of the soil columns); Distribution profiles of oocysts in soils (number of oocysts per gram of dry soil vs. depth of soil).

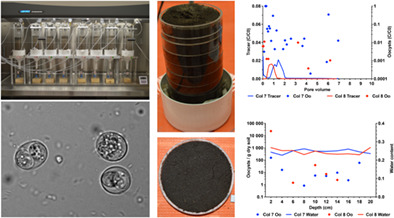

## INTRODUCTION

*Toxoplasma gondii* is one of the most prevalent parasites in warm‐blooded animals worldwide, including humans (Dubey, 2022; Robert‐Gangneux & Dardé, 2012). In all infected hosts, the parasite can multiply asexually in any nucleated cells and form latent cysts mainly in cerebral, muscular and ocular tissues (Dubremetz & Ferguson, 2009). In contrast, felids are the only natural hosts known to support the sexual multiplication of the parasite (Di Genova et al., 2019). It occurs within their intestinal epithelium and results in the production and faecal excretion of millions of an environmentally resistant form called the oocyst. After sporulation, oocysts measure ~12 × 11 μm and contain eight infectious sporozoites, which are protected from the environmental conditions by the oocyst and sporocyst walls (Freppel et al., 2018). The oocyst wall is highly proteinaceous with a lipid coating. It has a negative surface charge that likely contributes to the diffusion of the oocysts in fresh waters (Shapiro et al., 2009).

Oocysts ingested accidentally with soil particles, fresh products and waters contaminated by cat faeces can lead to *T. gondii* infections in humans and animals, sometimes as outbreaks (Pinto‐Ferreira et al., 2019; Shapiro et al., 2019; Torrey & Yolken, 2013). In turn, infected animals are important reservoirs of transmission for people eating raw or undercooked meat that may contain infectious cysts (Tenter et al., 2000). It has been estimated that about one third of the global human population is infected by the parasite (Montoya & Liesenfeld, 2004; Robert‐Gangneux & Dardé, 2012). The respective part of oocyst‐ and tissue cyst‐associated contamination routes in human infection is overall unknown, however. It appears to differ across geographical areas regarding the diet habits and the capacity of oocysts to remain infectious throughout the environment in conjunction with climatic conditions (Robert‐Gangneux & Dardé, 2012). Depending on the parasite genetic background and host immune status, *T. gondii* infections in humans may range from asymptomatic forms to severe ocular or cerebral diseases (Mukhopadhyay et al., 2020). Congenitally infected children and immunocompromised people (e.g., AIDS or grafted patients) are the main groups at risk of symptomatic, even severe toxoplasmosis (Robert‐Gangneux & Dardé, 2012).

Soils where cats deposit their faeces are the primary reservoirs of oocysts before the parasite spreads in the environment (Shapiro et al., 2019). The habit of felids to bury their faeces results in the incorporation of oocysts into the first few centimetres from the soil surface. This process could help protect the oocysts from inactivation by solar UV radiations, large temperature variations and desiccation (Aramini et al., 1999; Frenkel et al., 1975; Frenkel & Dubey, 1973). Moreover, soil macrofauna, such as coprophagous insects and earthworms, can passively transport oocysts on their tegument or in their digestive tract, and contribute to some extent in spreading the oocysts in soils, and plants in contact with soils (Frenkel et al., 1975; Ruiz & Frenkel, 1980; Smith & Frenkel, 1978). In addition to biotic factors, soil composition and moisture can greatly impact on the persistence of infectious oocysts in soils and, in conjunction with rainfall intensity, to their horizontal and vertical transport within soils and their subsequent transfer to surface and ground waters (Shapiro et al., 2019). Oocyst excretion behaviour of cats, oocyst incorporation pathways and abiotic factors likely result in a heterogeneous distribution of the parasites in soils, making uncertain the detection and characterization of the oocysts in such matrices and the modelling of oocyst transmission to humans and animals (Afonso et al., 2008). To date, *T. gondii* oocysts have been isolated, mainly as DNA, from naturally infected soils in different countries, but with little evidence of their infectivity (Shapiro et al., 2019). Despite the continuous improvement of oocyst detection methods (de Wit et al., 2020; Deng et al., 2021; Escotte‐Binet et al., 2019; Lass et al., 2009; Lélu et al., 2011; Wang et al., 2014; Wu et al., 2017), there is still a profound lack of knowledge about the mechanisms governing the persistence and spatiotemporal distribution of oocysts by soil type and use (Gao et al., 2016; Gotteland, Gilot‐Fromont, et al., 2014; Gotteland, McFerrin, et al., 2014; Lélu et al., 2012). In this context, the objective of this study was to characterize the transport and retention of *T. gondii* oocysts in natural soils with varying physicochemical properties and to investigate the effect of surfactant on the mobility of the oocysts.

## EXPERIMENTAL PROCEDURES

### Study design

To study the influence of soil physicochemical properties, water chemistry and surfactant on the fate, and transport of *Toxoplasma gondii* in soil water systems, columns containing either loamy sand or sandy loam soils were subjected to a simulated rainfall and addition of Aerosol 22, a widely used anionic surfactant that is present in many household cleaning products and which may enter soil through greywater for irrigation (Al‐Jayyousi, 2003; Hamilton et al., 2007). Comparisons of the oocyst transport behaviour in the various soil types were achieved with breakthrough curves (BTCs) and distribution profiles of oocysts from the soil columns following the artificial rain. Oocysts were detected and quantified by qPCR in both soil and leachate samples from all columns.

### Toxoplasma gondii *oocysts*

Viable *Toxoplasma gondii* oocysts of the ME 49 strain were obtained from faeces of cats at the United States Department of Agriculture Agricultural Research Service (USDA‐ARS) Beltsville Agricultural Research Center (BARC), Beltsville, Maryland, United States, as described by Dubey (1995) before the redirection of *Toxoplasma* research at the USDA, using approved protocols. Oocysts were cleaned of faecal debris by sugar flotation and by caesium chloride gradient (Dubey, 2010). Oocysts were sporulated in aqueous 2% H_2_SO_4_ by aeration at room temperature for 7 days, enumerated in a counting chamber by microscopy, and then stored at 4°C. Sporulated oocysts were transported from Beltsville, Maryland to Clemson, South Carolina by air, and from South Carolina, USA to France by air. The oocysts stock suspension was kept at 4°C prior to use. Prior to addition to the pulses applied to the columns, oocysts were vortexed at fixed speed of 3000 rpm for 5 min to ensure a uniform distribution of oocysts throughout the oocyst stock suspension.

### Soils

The soils used in this study were collected as bulk samples from fallow and cultivated fields in Illinois and Utah, USA (Darnault et al., 2017; Darnault et al., 2022). Soils were air dried at 37°C, sieved in a 2 mm sieve and stored at room temperature in lidded buckets until used. Their physiochemical properties, presented in Table 1, were determined by the Utah State University Analytical Laboratories (USUAL) following procedures described in detail by Darnault et al. (2017, 2022). The taxonomic classification of these soils and their series descriptions are also presented in detail in Darnault et al. (2017, 2022). Briefly, soil from the Gilford series, a loamy sand, was sampled in Kankakee County, Illinois. The taxonomic soil classification of the Gilford series is coarse‐loamy, mixed, superactive, mesic Typic Endoaquolls. The second loamy sand soil, the Sparta series, was also sampled from Kankakee County, Illinois. Its taxonomic classification is sandy, mixed, mesic Entic Hapludolls. Soil from the Greenson series, a sandy loam, was collected in Cache County, Utah. The taxonomic soil classification of the Greenson series is fine‐silty, mixed, superactive, mesic Oxyaquic Calcixerolls. The second sandy loam, the Lewiston series, was also acquired in Cache County, Utah. The taxonomic class of the Lewiston series is coarse‐loamy, mixed, superactive, mesic Aquic Calcixerolls.

**TABLE 1:** Physicochemical properties of the four series of soils examined—Sparta, Lewiston, Gilford and Greenson.

		Soil series			
		Sparta	Lewiston	Gilford	Greenson
Soil Texture	Sand (%)	82.00	79.70	84.40	66.90
	Silt (%)	8.40	7.70	7.70	13.60
	Clay (%)	9.50	12.50	7.90	19.50
	USDA texture	LS	SL	LS	SL
pH		6.90	7.50	5.20	7.40
Soil Carbon	TC (%)	1.93	1.28	2.91	2.00
	TOC (%)	1.93	1.28	2.91	2.00
Organic Material (%)	Walkey‐Black	2.80	0.60	3.30	2.80
	Loss on Ignition	3.40	0.80	4.20	3.80
Total Nitrogen (%)		0.15	0.13	0.19	0.08
EC (dS m^−1^)		0.36	1.01	0.68	0.75
CEC (mmol_c_ kg^−1^)		86	115	84	175
Elements and Ions	Sodium (mg kg^−1^)	3.27	11.29	1.87	13.55
	Potassium (mg kg^−1^)	13.80	22.00	26.70	10.80
	Magnesium (mg kg^−1^)	4.84	14.91	5.71	12.00
	Calcium (mg kg^−1^)	9.36	37.10	14.64	42.09
	Chloride (mg L^−1^)	10.40	53.20	44.60	49.90
	Sulfur (mg kg^−1^)	1.79	7.50	3.73	8.40
	Nitrate‐N (mg kg^−1^)	12.60	0.40	44.10	0.90
	Boron (mg kg^−1^)	0.02	0.04	0.02	0.02
	Carbonate + Bicarbonate (mmol_c_ L^−1^)	1.80	8.41	0.50	6.04

*Note*: Adapted from Reference Darnault et al. (2017). Copyright © 2017 Darnault, Peng, Yu, Li, Jacobson and Baveye. This is an open‐access article distributed under the terms of the Creative Commons Attribution Licence (CC BY).

Abbreviations: CEC, cation exchange capacity; EC, electrical conductivity; LS, loamy sand; SL, sandy loam; TC, total carbon; TOC, total organic carbon.

### Artificial rainfall and oocyst inoculum solutions

All solutions were prepared using deionized water (DI) water. The rainfall solutions contained 1 mM potassium chloride (KCl). The rainfall was continuously applied to the columns at a velocity of about 1 cm hr^−1^. A conservative tracer, potassium bromide (KBr), was used and applied with the oocyst inoculum at a concentration of 10 mM. The conservative tracer KBr was measured in the inoculum and leachate samples using an Orion™ Bromide Electrode (ThermoFisher Scientific, Waltham, Massachusetts, USA). The oocyst inoculum suspension was prepared by adding 1 mL of the oocysts stock suspension to 49 mL of the 1 mM KCl artificial rainfall solution that was spiked with 10 mM KBr tracer. 45 mL of the inoculum were applied to the columns, while 5 mL was retained to quantify the initial concentration (C_0_) of oocysts. In two columns of each soil type, the artificial rainfall also contained 2 critical micelle concentration (CMC) of the surfactant Aerosol 22 (Sigma‐Aldrich, St. Louis, MO, USA). The inoculum applied to the soil surface of each column contained a concentration of *T. gondii* oocysts of about 18,877 mL^−1^ (quantified and enumerated from the oocyst stock suspension using the qPCR method), corresponding to a total of 849,487 oocysts.

### Column and rainfall simulator

The columns used in this study were made of plastic rings and were 20 cm in length with a 9.5 cm internal diameter. A 5 cm ring in height was placed at the base of the column atop a mesh fabric and a screen to prevent soil loss. A total of 2 cm rings in height were then mounted on the 5 cm ring to produce a height of 21 cm. Then, one and 2 cm rings were then placed on top of the base ring to achieve a total height of 30 cm, 20 of which were later filled with soil. To enhance stability, the rings were compressed between the top and bottom plastic column holders with four rods positioned parallel to the length of the column, which were bolted to the top and bottom sections. A funnel was clamped separately to the bottom column holder. An additional rod was used behind the column to attach a misting nozzle about 10 cm above the soil surface.

Soil was packed into each column at a uniform bulk density of 1.53 g cm^−3^. A soil mass of 2125 g was packed into each column in three equal amounts and after each addition the soil was compacted with a rod.

Eight flow‐through experimental columns were run simultaneously. A peristaltic pump (Cole‐Parmer, Vernon Hills, IL) was fitted with a multichannel cartridge pump head that held eight tubes to pump the artificial rainfall solution into the misting nozzles that were attached above each column. The misting nozzles (XA nozzle system 1/4, 303 from BETE Fog 218 Nozzle Inc., Greenfield, MA) were connected to the pump on one side with brass couplings and to air on the other. The flow rate through the nozzles was adjusted using Parker Watts miniature precision regulator gauge (1/4 in; 60 psi; Parker Hannifin Corp., Cleveland, OH) to create a mist that covered the entire soil surface.

### Experimental procedure: Rainfall treatments and leachate collection

Once columns were filled with soil and placed in the stands, the artificial rainfall was started. Outflow from each column was observed until it reached steady state, at which point the rainfall was stopped and the oocyst inoculums were added to the surface. The pulses were allowed to infiltrate fully before the rainfall was resumed. Leachate samples were collected in varying volumes throughout the experiment using 50 mL conical‐bottom polypropylene centrifuge tubes for the first 24 h and beakers for greater volumes after this period or roughly two pore volumes (PVs). For samples exceeding 50 mL, their volumes were recorded, and the samples were mixed with a magnetic mixer and 50 mL subsamples were taken. After at least six PVs had been collected from each column, rainfall was shut off.

### Soil water content

Each column was sliced into 2 cm slices for analysis of soil water content and oocyst concentration. Soil water content was determined by taking two 5 g samples from each soil layer. The weight of these samples was recorded and they were placed in aluminium foil cups and dried at 105°C in an oven for 24 h. The dry weight was recorded, and water content was determined gravimetrically.

### Isolation of oocysts from soils

Following the removal of water content samples, each soil layer was mixed thoroughly to distribute oocysts evenly throughout the soil. Two soil samples were taken from the mixed soil layer. Each replicate weighed approximately 25 g and the mass of the sample was recorded. Each sample was placed in a 50 mL centrifuge tube. The method developed by Koken et al. (2013) was then used to isolate oocysts from the soil. To release oocysts from the soil, 20 mL of a solution containing Tween 80 at 2 critical micelle concentrations (CMC) and 50 mM TRIS buffer was added to each tube (Koken et al., 2013). The centrifuge tubes were then attached to a rotational shaker, perpendicular to the axis of rotation. Following shaking for 24 h, the tubes were centrifuged at 2500*g* for 15 min. The resulting supernatant was transferred to a new 50 mL conical‐bottomed centrifuge tube and centrifuged again at 2500*g* for 15 min. The supernatant was removed to leave 4 mL in the tube. For the first wash, the remaining 4 mL was vortexed and 35 mL of deionized water was added to the tube to resuspend the pellet. A second wash was completed following the procedure of the first wash. For the third wash, the remaining 4 mL were vortexed and transferred into 15 mL centrifuge tubes.

### Concentration of oocysts in leachates

The method developed by Koken et al. (2013) was used to isolate oocysts from leachate, with every second sample evaluated for oocyst concentration. Samples were centrifuged at 2500*g* for 15 min to concentrate oocysts in the pellet. The supernatant was removed and discarded, leaving 5 mL in the centrifuge tube. The remaining pellet was vortexed for 20 s and 1 mL of the mixed sample was transferred into a microcentrifuge tube for oocyst quantification.

### DNA extraction and qPCR analysis of oocysts

For DNA extraction, soil and leachate samples resulting from the processes of isolation of oocysts from soils and concentration of oocysts in leachates were centrifuged at 10,000*g* for 5 min to concentrate oocysts in a 50 μL sample. Samples were then subjected to six cycles of freezing at −80°C for 5 min and thawing at 90°C for 5 min prior to sonication for 10 min in a sonicator bath to weaken the oocyst and sporocyst wall. DNA was extracted with the E.Z.N.A.® Tissue DNA kit (OMEGA Bio‐Tek, #D3396‐02, VWR International S.A.S., Strasbourg, France) in accordance with the manufacturer's instructions, except that DNA was eluted in 70 μL pre‐warmed TE buffer. The real‐time PCR assay targeted the 529 bp repeat region (REP529, GenBank accession no. AF487550) of *T. gondii* (Reischl et al., 2003). Real‐time PCR reactions were performed on a Roche's LightCycler 480 in a final volume of 25 μL containing 1× LightCycler™ 480 Probes Master (ROCHE Diagnostics, France), 0.5 μmol/L of each primer, 0.25 μmol/L of the Taqman probe, 0.5 μL of 1% bovine serum albumin (BSA) and 2 μL of template DNA. The nucleotide sequences of the primers were 5′‐AGG AGA GAT ATC AGG ACT GTA G‐3′ and 5′‐GCG TCG TCT CGT CTA GAT CG‐3′. The nucleotide sequence of the Taqman probe was 5′‐6‐ FAM‐ CCG GCT TGG CTG CTT TTC CTG‐ TAMRA‐3′. The Taqman probe and nucleotide sequences were purchased by EUROGENTEC (France). Thermal cycling conditions were 95°C for 10 min, 45 cycles of 10 s at 95°C, 30 s at 58°C and 10 s at 72°C. Results were expressed in cycle threshold values (Ct) and the oocyst number was derived using a standard curve created by five, 10‐fold serial dilutions of a sample with an oocyst concentration determined by an enumeration of the stock oocyst batch (178,571 oocysts/μL). All DNA samples were tested in duplicate and each assay was considered positive if at least one of the duplicated tests was positive. Each PCR run included a positive control and a negative control with no DNA.

### Mathematical analysis

The mathematical analysis of flow and transport through porous media experiments or chemical reactors can be performed using the moments of a function. Here, quantitative measures are used related to the shape of the graph of the function, for example, the BTCs. Three moments are usually calculated for the mathematical analysis of BTCs to characterize the mean residence time, the variance and the skewness of the results. The first moment, or mean residence time, is defined by the equation:

(1) $$t_{m}=\int_{0}^{\infty }\mathrm{tE}\left(t\right)\mathrm{dt}$$

Equation 1 describes the mean residence time in the reactor between time t and t+∆t for particles (e.g., oocysts), solute, or tracer within the column reactor using the E curve which is described as:

(2) $$E\left(t\right)=\frac{C\left(t\right)}{\int_{0}^{\infty }C\left(t\right)\mathrm{dt}}$$

The E curve equation describes the concentration C of the particles (e.g., oocysts) and tracer with respect to time t over the integral of the concentration of oocysts and tracer with respect to time.

The second moment, or the variance or square of the standard deviation, is taken about the mean of the residence time. It is defined by the equation:

(3) $$\sigma^{2}=\int_{0}^{\infty }{\left(t-t_{m}\right)}^{2}E\left(t\right)\mathrm{dt}$$

The magnitude of this moment is an indication of the “spread” of the distribution; the greater the value of this moment, the greater the spread of the distribution.

The magnitude of the second moment indicates the spread of the distribution of the breakthrough concentrations. Larger second moments indicate a greater distribution of the spread of results from the mean residence time of the particles (e.g., oocysts) or tracer within a system.

The third moment is also measured about the mean residence time, and it represents the skewness of the data. It is defined by the equation:

(4) S3=1σ32∫0∞t−tm3Etdt

The third moment measures the level at which a distribution is skewed in one direction or another with respect to the mean distribution of the BTCs. A positive value for the skewness will result in a tailing effect of the BTC and is the expected result for flow and transport processes which react with the porous media.

## RESULTS

### Soil physicochemical characteristics

The Greenson and Lewiston series were characterized as sandy loam soils, and the Gilford and Sparta series as loamy sand soils (Table 1). The clay contents in the sandy loam soils were the highest with Greenson containing 19.5% clay and Lewiston containing 12.5% clay. The organic matter contents derived from Loss on Ignition (LOI) analysis of the soils were greatest in the Gilford (4.2%) and Greenson (3.8%) soil series. The highest pH values were found in Lewiston (7.50) and Greenson (7.40) while the lower pH values were found in the Sparta (6.90) and Gilford (5.20) soil series. Total calcium concentrations were the lowest in Sparta at 9.36 mg kg^−1^ and the highest in Greenson at 42.09 mg kg^−1^.

Both the flow rate and flow velocity for each experimental column are provided in Table 2. The highest average flow velocity was observed in Sparta soils that did not contain surfactant (0.52 cm hr^−1^). The lowest average flow velocity was observed in Greenson soil containing surfactant (0.29 cm hr^−1^).

**TABLE 2:** Flow rate and flow velocity for each of the four series of soils examined—Sparta, Lewiston, Gilford and Greenson.

Soil type	Surfactant	Column #	Flow rate (mL hr^1^)	Average flow rate (mL hr^−1^)	Flow velocity (cm hr^−1^)	Average flow velocity (cm hr^−1^)
Sparta	No	1	35.25	37.04	0.50	0.52
		2	38.83		0.55	
	Yes	3	36.00	35.89	0.51	0.51
		4	35.78		0.50	
Lewiston	No	5	35.62	33.92	0.50	0.48
		6	32.21		0.45	
	Yes	7	29.58	27.71	0.42	0.39
		8	25.83		0.36	
Gilford	No	9	29.95	30.25	0.42	0.43
		10	30.55		0.43	
	Yes	11	36.40	30.94	0.51	0.44
		12	25.48		0.36	
Greenson	No	13	32.84	32.87	0.46	0.46
		14	32.89		0.46	
	Yes	15	17.43	20.58	0.25	0.29
		16	23.73		0.33	

### Prevalence and concentration of oocysts in soil leachates

**FIGURE 1:**
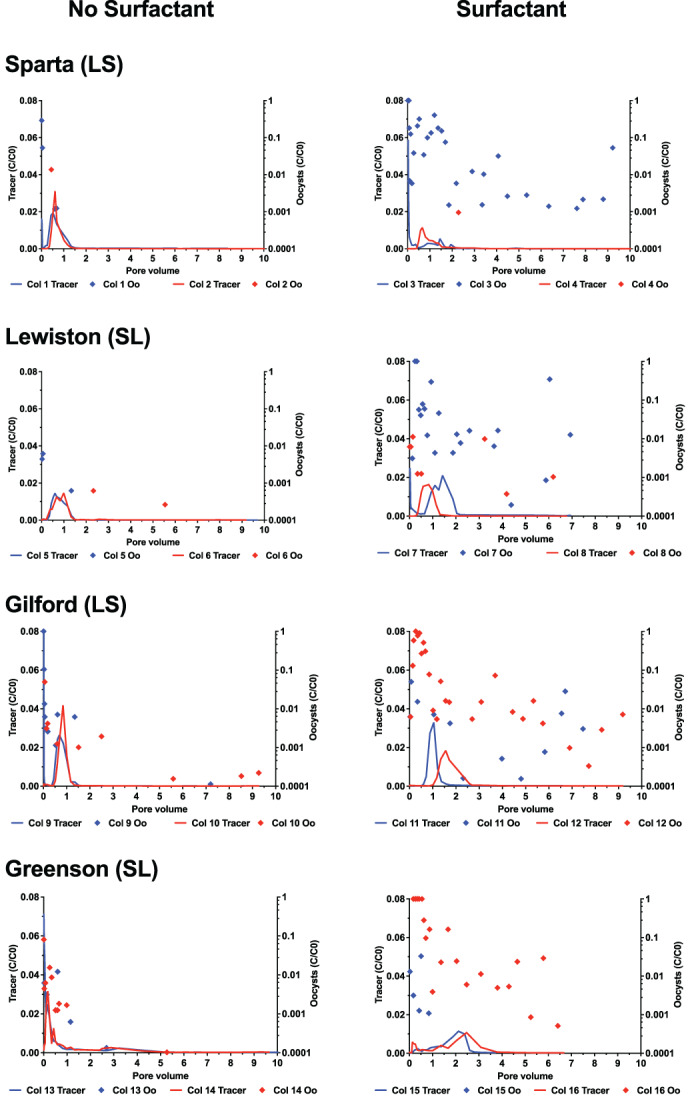
Breakthrough curves (BTCs) of *Toxoplasma gondii* oocysts and bromide tracer in loamy sand and sandy loam soils from the four series of soils examined (Sparta, Lewiston, Gilford, and Greenson) during simulated rainfall in the absence and presence of Aerosol 22 surfactant. C, concentration of *T. gondii* oocysts (oocysts mL^‐1^) or Br^−^ (mg L^−1^) in each leachate sample; C_0_, concentration of *T. gondii* (oocysts mL^−1^) or Br^−^ (mg L^−1^) inflowing the soil column. Oo, Oocysts; LS, loamy sand; SL, sandy loam. For ease of data visualization, the right y axis is log10‐scaled.

Leachate samples were collected from at least 6 PVs of each soil column. The number of oocysts in the leachates from two replicates of the four soils with and without surfactant are represented in breakthrough curves (BTCs) as C/C_0_, where C is the concentration of oocysts detected in the leachates and C_0_ is the concentration of oocysts on average in the input solutions (Figure 1). The calculation of C_0_ excluded the pulses of the columns where no oocyst was detected (column #7). The C_0_ value of the pulse sample of column 7 was excluded from the calculation of the C_0_ value (concentration of oocysts on average in the input solutions) due to a non‐amplification of the pulse sample of column 7. For detection of oocysts where C/C_0_ exceeded 1, as a result of the C_0_ value being obtained from averaging all C_0_ values, values were reduced and instead plotted as 1.

In the Sparta soil series without surfactant (columns #1 and #2), oocysts were only detected within the first PV of leachate in minimal concentrations (Figure 1). The peaks of BTCs of tracer occurred at 0.51 and 0.61 PV, and the peaks of the tracer BTCs measured as C/C_0_ ranged from 0.019 to 0.031. Oocysts peaked in columns #1 (C/C_0_ value of 0.29 at 0.008 PV) and #2 (C/C_0_ value of 0.014 at 0.43 PV) at early PVs of leachate compared to the tracer (Figure 1). In the Sparta soil containing surfactant (column #3 and 4), oocysts were detected in much greater concentrations in replicate 1 (column #3) than in replicate 2 (column #4). Oocysts peaked in column #3 at early PVs of leachate (Figure 1). Peaks of oocysts BTCs reached values of 1 from 0.01 to 0.05 PVs in column #3 and 0.001 at 2.29 PV in column #4. Peaks of tracer BTCs occurred at 0.02 PV with a C/C_0_ of 0.058 in column #3 and at 0.66 PV with a C/C_0_ of 0.011 in column #4.

In the Lewiston soil series without surfactant (columns #5 and #6), oocysts were detected at low concentrations in both replicates—from 0.0006 to 0.006 in column #5, and from 0.0002 to 0.0006 in column #6). Tracer peaked at 0.60 and 0.99 PV. The maximum peak value of tracer BTCs was 0.015 in both columns (Figure 1). Oocysts peaked in column #5 at early PVs of leachate compared to the tracer (Figure 1). In the Lewiston soil containing surfactant (columns #7 and #8), oocysts were detected throughout the experiments in both columns. In the column #7, the greatest concentrations of oocysts appeared at 0.28–0.32 PV, with C/C_0_ peak values reaching 1. Column #7 displayed an early tracer peak at 0.008–0.024 PVs with C/C_0_ peak values of 0.024–0.025, and another peak at 1.43 PV with a C/C_0_ peak value of 0.021. In the column #8, the peak of oocysts BTC appeared at 0.15 PV, with C/C_0_ peak values reaching 0.01. The tracer peak in column #8 occurred at 0.83 PV and had a C/C_0_ peak value of 0.016. Oocysts peaked in columns #7 and 8 at early PVs of leachate compared to the tracer (Figure 1).

In the Gilford soil series without surfactant (columns #9 and #10), oocysts were mainly detected in several samples at early times. BTCs of oocysts showed C/C_0_ peak values of 1 at 0.008 PV (column #9) and 0.05 at 0.06 PV (column #10). In column #9, the tracer peaked at 0.008 PV with a C/C_0_ value of 0.11. Column #10 had a tracer peak at 0.85 PV with a C/C_0_ value of 0.04. In Gilford soils containing surfactant (columns #11 and #12), oocysts were detected in numerous leachate samples throughout the experiment in both columns at concentrations higher than that observed in columns without surfactant. The BTCs of oocysts depicted peak values of C/C_0_ of 0.05 and 1 at 0.078 and 0.27 PVs for column #11 and #12, respectively. The tracer peak in columns #11 and #12 occurred at 1.05 PV with a C/C_0_ value of 0.03, and at 1.56 PV with a C/C_0_ value of 0.02, respectively.

In the Greenson soil series without surfactant (columns #13 and #14), oocysts were detected mostly in samples at early times. BTCs of oocysts showed C/C_0_ peak values of 0.01 at 0.59 PV (column #13) and 0.08 at 0.008 PV (column #14). In column #13, the tracer peaked at 0.008 PV with a C/C_0_ value of 0.07. Column #14 had a tracer peak at 0.178 PV with a C/C_0_ value of 0.03. The BTCs of oocysts depicted peak values of C/C_0_ of 0.013 and 1 at 0.026 and 0.17–0.55 PVs for columns #15 and #16, respectively. The tracer peak in columns #15 and #16 occurred at 2.11 PV with a C/C_0_ value of 0.01, and at 2.47 PV with a C/C_0_ value of 0.01, respectively.

### Spatial distribution of oocysts in soils

**FIGURE 2:**
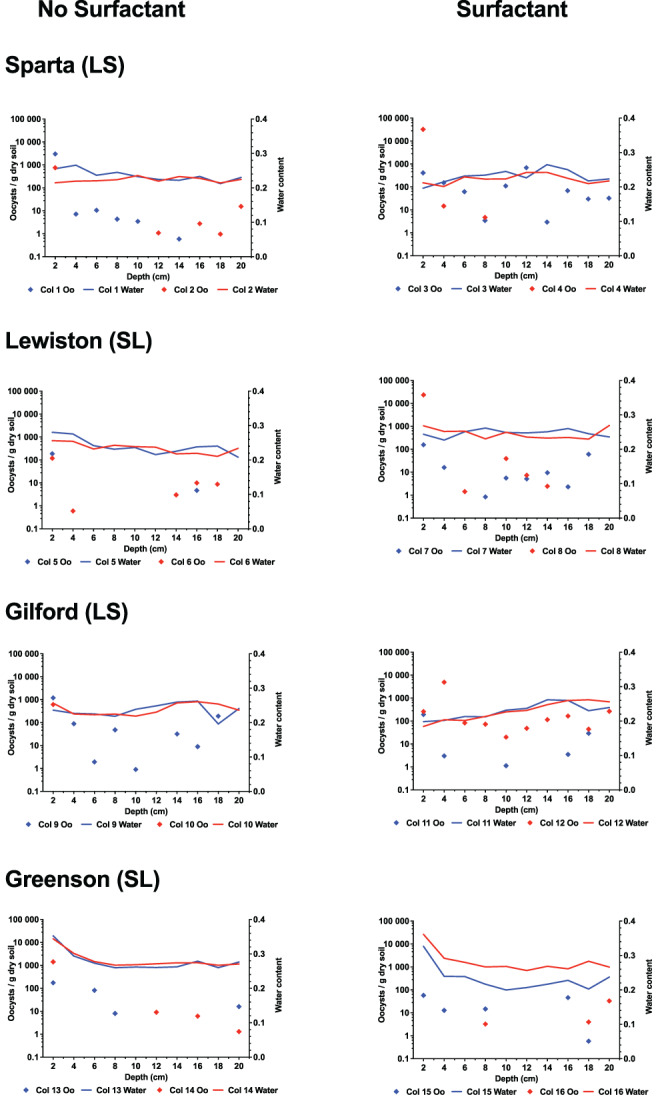
Spatial distribution of *Toxoplasma gondii* oocysts recovered from soil columns and volumetric water content in soil profiles in loamy sand and sandy loam soils from the four series of soils examined (Sparta, Lewiston, Gilford and Greenson) following the application of rainfall treatments in the absence and presence of Aerosol 22 surfactant, and about six pore volumes. Oo, Oocysts; LS, loamy sand; SL, sandy loam. For ease of data visualization, the left y axis is log10‐scaled.

Following the flow and transport experiments, soil columns were sliced into layers and subsampled in duplicates. They were then analysed to determine the concentrations and spatial distribution of oocysts and water content throughout each column (Figure 2).

In the Sparta soil (columns #1–4), oocysts concentrated near the top of the columns in both the presence (columns #3–4) and absence of surfactant (columns #1–2; Figure 2). In both replicates, the concentration of oocysts was highest in the first soil layer. In Sparta soil without surfactant, the concentration ranged from 754 to 3005 oocysts/g of dry soil (column #2 and #1, respectively). The distribution of oocysts in surfactant‐containing Sparta soil columns differed from that of columns without surfactant. In column #3, oocysts were detected throughout the soil column, with the greatest concentration (690 oocysts/g) at about 12 cm from the surface, and a concentration of 410 oocysts/g of dry soil in the first soil layer. In column #4, concentrations were higher at lower depths of the soil column and comparatively lower at greater depths. Oocyst concentration peaked in the first layer and was 32,364 oocysts/g of dry soil.

In the Lewiston soil series (columns #5–8), oocysts were concentrated in the uppermost soil layers in those columns with (columns #7–8) or without surfactant (columns #5–6; Figure 2). The maximum concentration of oocysts was 188 and 119 oocysts/g of dry soil in columns #5 and #6 respectively. In columns with surfactant, the maximum concentration of oocysts was 157 and 23,439 oocysts/g of dry soil in the first top layer of columns #7 and #8 respectively.

In the Gilford soil series (columns #9–12), concentrations of oocysts are highest at shallow depths in columns that either did (columns #11–12) or did not contain surfactant (columns #9–10; Figure 2). In columns #9–10, oocyst concentrations were greatest in the uppermost layers of soil and peaked at 1194 and 616 oocysts/g of dry soil respectively. In column #11, the maximum concentration was about 192 oocysts/g of dry soil and occurred in the uppermost layer of soil. In column #12, the maximum concentration was 1942 oocysts/g of dry soil and occurred in the second layer of soil.

In the Greenson soil series (columns #13–16), concentrations of oocysts were greatest in the uppermost layer of soil without surfactant (columns #13–14; Figure 2). The maximum concentrations were 175 and 1418 oocysts/g of dry soil in columns #13 and #14 respectively. In columns with surfactant, oocysts were distributed through depth homogeneously. The maximum concentrations were 58 oocysts/g of dry soil in the first soil layer of column #15 and 33 oocysts/g of dry soil in the deepest soil layer in column #16.

### Recovery of oocysts in leachates and soils

Table 3 gives the recovery results and predominant fate of oocysts in each soil column, in both the absence and presence of surfactant. In the absence of surfactant, the predominant fate of oocysts in soils subject to simulated rainfall was their retention in the soil profiles, with 7 out of the 8 soil columns tested (87.5%) showing a higher retention of oocysts in the soil than their presence in the soil leachates. The presence of surfactant induced a change in the fate of oocysts in these soils exposed to rainfall simulation as the predominant fate of oocysts was found to be in the soil leachates, indicating their breakthrough, compared to their retention in the soil profiles. Out of the 8 soil columns tested, 6 soil columns (75%) exhibited a higher breakthrough of oocysts than their retention in soil.

**TABLE 3:** Recovery data for *Toxoplasma gondii* oocysts in leachate and soil for each column.

Soil USDA texture	Column	Theoretical number of oocysts in pulses	Total number of oocysts detected in leachate	Total number of oocysts detected in leachate (%)	Total number of oocysts detected in soil	Total number of oocysts detected in soil (%)	Total oocysts recovery (%)	Predominant fate
Sparta Loamy sand	1	849,487	39,866	4.69	472,587	55.63	60.33	SOIL
	2	849,487	12,952	1.52	187,168	22.03	23.56	Soil
	3	849,487	17,572,242	2068.57	315,198	37.10	2105.68	Leachate
	4	849,487	7307	0.86	7,774,378	915.19	916.05	Soil
Lewiston Sandy loam	5	849,487	3342	0.39	25,768	3.03	3.43	Soil
	6	849,487	6246	0.74	23,557	2.77	3.51	Soil
	7	849,487	6,572,196	773.67	70,948	8.35	782.02	Leachate
	8	849,487	29,873	3.52	5,716,660	672.95	676.47	Soil
Gilford Loamy sand	9	849,487	165,992	19.54	271,882	32.01	51.55	Soil
	10	849,487	22,172	2.61	132,828	15.64	18.25	Soil
	11	849,487	194,544	22.90	53,435	6.29	29.19	Leachate
	12	849,487	5,942,361	699.52	718,490	84.58	784.10	Leachate
Greenson Sandy loam	13	849,487	68,084	8.01	55,795	6.57	14.58	Leachate
	14	849,487	40,640	4.78	338,275	39.82	44.61	Soil
	15	849,487	54,929	6.47	27,285	3.21	9.68	Leachate
	16	849,487	186,495,967	21953.95	9742	1.15	21955.10	Leachate

*Note*: Grey shading indicates that a column contains surfactant.

## DISCUSSION

The diffusion/retention of *T. gondii* oocysts in soils and sediments critically affects the transmission of the parasite to humans and animals, from terrestrial to aquatic environments (Shapiro et al., 2019). The transport of oocysts in undisturbed soils and unsaturated porous media and in receiving surface and ground waters has been hypothesized as dependent upon the oocyst surface properties and soil characteristics in conjunction with rainfall intensity (Dumètre et al., 2012). In the present study, we aimed to more thoroughly elucidate the soil physicochemical properties that govern the movement and spatial distribution of oocysts in soil. Here, transport, and fate of oocysts in unsaturated soils of different types were investigated using repacked and homogeneous natural soil columns subject to simulated artificial rainfalls, both with and without surfactant. Overall, oocysts were detected in all soil types (loamy sand, sandy loam). In most cases, the parasites were detected in highest concentrations in upper layers of soil, typically within the first 2–4 cm depth. Although oocyst transport via infiltrating water through the soil column was demonstrated in all soils, it differs between soils and whether surfactant was applied to columns.

In the absence of surfactant, straining and adsorption are the central criteria controlling the transport of the parasites throughout the soil column as observed for other microorganisms (Bashan & Levanony, 1988; Bitton et al., 1974; Bradford & Bettahar, 2005; Darnault et al., 2017; Dumètre et al., 2012; Huysman & Verstraete, 1993; Mawdsley et al., 1996; Tan et al., 1991). Both soil grain size and grain shape play an important role in the extent of straining (Tufenkji et al., 2004). Increased grain size has been shown to increase straining. Although physical straining is thought to play a significant role in particle retention at particle diameter‐to‐median soil grain (DP/D50) sizes greater than 0.05, the importance of such straining is also evident with a DP/D50 as low as 0.002 (Tufenkji, 2007). Straining has been identified as an important mechanism for the retention of *C. parvum* oocysts in soil by Tufenkji et al. (2004). Therefore, given that *T. gondii* oocysts are larger in size (11–13 μm) than *C. parvum* (4–6 μm), straining is likely a factor controlling the retention of *T. gondii* oocysts in soils, and their subsequent removal from leachates.

Adsorption of *T. gondii* oocysts to soil grains does depend upon both oocyst surface properties and soil chemistry. The molecular coverage of oocysts renders them hydrophilic and negatively charged (Shapiro et al., 2019), which likely cause repulsive forces between the parasites and soil particles (i.e., clay, silt and sand) and consequently their transport throughout the soil columns. In addition, both the organic matter (OM) and the clay content of soil are particularly influential on the adsorption of microorganisms because of their large surface area and negative charge (Reddy et al., 1981). Here, enhanced transport was observed in both Gilford and Greenson soils compared to other soil series (Lewiston and Sparta soils) studied without surfactant. Gilford is a loamy sand and Greenson a sandy loam, indicating that the soil type may not be the primary factor controlling the transport of *T. gondii* oocysts in our study. However, both soils contain the greatest amount of OM as compared to other soils in the series. The presence of OM has been shown to increase cation exchange capacity, surface area and number of sites for bacterial adsorption (Zhong et al., 2017). An increase in OM has been found to cause a decrease in collision efficiency of sand and *C. parvum* oocysts, which produces a higher degree of breakthrough (Abudalo et al., 2010). Whether a similar mechanism occurs with *T. gondii* oocysts, however, requires additional experiments using, for instance, engineered sandy soils spiked with organic compounds of different natures and concentrations.

The inclusion of the anionic surfactant Aerosol 22 promoted transport of oocysts in all soil types. In several columns, oocysts were detected at lower depths indicating that considerable transport occurred. This was particularly the case for the Greenson soil samples containing surfactant. Many surfactant‐containing columns exhibited tailing concentrations throughout the duration of the experiments. A slight decrease was observed in flow velocity in Sparta, Lewiston, and Greenson soils, caused by the surfactant when the averages of replicates were compared. Inconsistent velocity was also noted between the columns of the same soil type and the surfactant status. Indeed, decreases in flow velocity with the addition of surfactant may be attributed to the clogging as indicated in the study of Mingorance et al. (2007). Here, the addition of Aerosol 22, which precipitates as salts and lowers the soil system porosity, resulted in an increase in saturated hydraulic conductivity (Ks) in silty clay loam soil and a decrease in Ks in both clay soil and silty loam soil. Further, transport of Aerosol 22 solutions through soil was affected by Ca^2+^ and Mg^2+^ cations in the soil (Mingorance et al., 2007). Peng et al. (2017) also noted an overall decrease in Ks in sandy loam and loamy sand soils studied in saturated soil column experiments under the influence of surfactant, though significant variation in Ks did occur over time and throughout the various soil layers. The variations in Ks were hypothetically attributed to the swelling of clay, the collapse of soil aggregates and subsequent particle displacements from surfactant adsorption. Particle displacement caused pore clogging in low layers and a higher porosity in the layers above (Peng et al., 2017).

Surfactants have been shown to enhance the transport of microbes through a variety of mechanisms. Steric forces may be produced when surfactants adsorb onto porous media and prevent microorganisms from approaching the porous media surface, reducing their interactions with soil particles (Brown & Jaffe, 2001; Zhong et al., 2017). The presence of steric forces due to the addition of surfactant may also contribute to enhanced transport in soil columns containing surfactant. In unsaturated systems, microbial transport is impacted by the presence of the air‐water interface (Wan et al., 1994; Zhong et al., 2017). Surfactants have been observed to reduce air‐water surface tension and allow for the wider spread of microorganisms through the system (Liu et al., 2011). Further, in their expansion of the electric double layer around bacteria and porous media, Brown and Jaffe (2001) observed a nonionic surfactant enhanced transport of a bacterial culture through porous media, which thus increased the electrostatic repulsion of materials and reduced collision frequency. Further, van der Waals and hydrophobic forces can serve as catalysts for surfactant adsorption onto cell surfaces, which causes a reduction in cell surface hydrophobicity and weakens adsorption by bacteria (Gorna et al., 2011; Zhang & Miller, 1994; Zhong et al., 2017). Anionic surfactants, such Aerosol 22, bring additional negative charge to cell surfaces, which decreases its zeta potential and weakens the interaction between bacteria and sand surface (Ishigami et al., 1987). Surfactant may also change the surface charge of soil particles. Indeed, an increase in the negative zeta potential of sand was observed with an increase in the concentrations of anionic surfactant, which enhanced bacterial transport (Bai et al., 1997; Chen et al., 2004; Zhong et al., 2017). In their demonstration of the influence of Aerosol 22 upon the fate and transport of *C. parvum* oocysts in soils Darnault et al. (2022) determined transport and retention of *C. parvum* oocysts in soil according to the physical and chemical parameters considered—soil texture, soil physics and chemistry, and surfactant. The result was a termination in either the enhancement or hinderance of *C. parvum* oocysts adsorption to soil particles, and their movement in soils. In our experimental system, Aerosol 22 might impact on the interactions of soil particles and *T. gondii* oocysts by inducing steric forces, thus reducing both the frequency of oocyst adsorption onto soil particles by both coating oocysts and soil particles; and the air‐water surface tension.

A comparison of the average values of the first moment of the mean residence time of *T. gondii* oocyst breakthrough in soils without surfactant corresponding to the column replicates shows that the averaged first moment values indicated a breakthrough of oocysts earlier in loamy sand soils compared to sandy loam soils (Table 4). Loamy sand soils exhibited $t_{m}$ values of 0.2 and 0.06 PV for Sparta series and Gilford series respectively, while sandy loam soils exhibited $t_{m}$ values of 2.05 and 5.46 PV for Lewiston and Greenson soil series, respectively. Analysis of the first moments of oocyst transport in the presence of surfactant demonstrated a delayed oocyst breakthrough in loamy sand soils and an enhanced breakthrough in sandy loam soils compared to the transport experiments without surfactant. In the presence of surfactant in loamy sand soils, the mean residence time of the oocyst breakthrough was increased to $t_{m}$ values of 1.6 and 3.35 PV for the Sparta and Gilford series; in sandy loam soils, the mean residence time decreased to $t_{m}$ values of 1.73 and 0.46 PV for the Lewiston and Greenson series. Comparison of the first moments for oocysts and tracer indicated an earlier breakthrough for oocysts compared to the tracer for loamy sand soils and a later breakthrough for oocysts compared to the tracer for sandy loam soils. In the presence of surfactant, the first moments show a later breakthrough of oocysts compared to the tracer for all soils, except for the Greenson soil series where an earlier breakthrough was observed. The observation of an early breakthrough of the oocysts in comparison with the breakthrough of the tracer indicates that oocyst transport is subject to an increase due to certain mechanisms. Previous studies investigating the fate and transport of bacteriophage MS2 in water saturated porous media under the influence of organic compounds and electrolytes exhibited a comparable behaviour (Keller et al., 2004). This observed transport behaviour for bacteriophage MS2 was attributed to size exclusion phenomena that resulted in the rapid transport of colloid. This size exclusion phenomena are primarily the result of the inability of colloids to enter the pores due to their size. Other possible mechanisms include the inaccessibility of a component of the pore and therefore inducing an earlier breakthrough (Chrysikopoulos & Katzourakis, 2015). The existence of an inaccessible pore space in soils to oocysts channels their transport via preferential pathways, resulting in an increase of their initial breakthrough in comparison with a nonreactive tracer. A comparison of the average second moment variance of the BTCs of the oocysts and tracer in soils yields no definite conclusions, however (Table 4). The third moment of skewness of the BTCs for oocysts were characterized by differences when comparing systems both with and without surfactant (Table 4). An increase in the third moments, usually observed for most soil columns, is the likely result of an inhibition of the transport of the oocysts through the system resulting in a tailing effect. Therefore, using the method‐of‐moments to analyse the fate and transport of oocysts in soils reveals the existence of an inaccessible pore space in soils to oocysts, resulting in their transport via preferential pathways, as well as subsequent mitigation of their transport.

**TABLE 4:** The values of moments—mean residence time (first moment), variance (second moment), and skewness (third moment)—of the observed effluent breakthrough curves (BTCs) for non reactive tracer and *Toxoplasma gondii* oocysts in different soil water systems.

Soil water systems	BTCs of tracer and *T. Gondii*	Mean residence time (first moment)	Variance (second moment)	Standard deviation	Skewness (third moment)
LS ‐ Sparta No Surfactant	BTCs of Columns 1 and 2				
	1 Tracer	0.54	0.116930848	0.341951528	115.4145612
	1 *T. gondii*	0.01	0.000406617	0.020164756	3.133286315
	2 Tracer	0.64	0.039435708	0.198584259	106.6315157
	2 *T. gondii*	0.40	4.68107E‐05	0.006841833	6.91785E‐05
	Average values of 1 & 2 Tracer	0.59	0.08	0.27	111.02
	Average values of 1 & 2 *T. gondii*	0.20	0.00	0.01	1.57
LS – Sparta Surfactant	BTCs of Columns 3 and 4				
	3 Tracer	0.91	0.049879891	0.223338065	72.16886197
	3 *T. gondii*	1.00	0.081719405	0.285866061	155.5332442
	4 Tracer	0.92	0.076339517	0.276296067	120.8988533
	4 *T. gondii*	2.25	4.68095E‐05	0.00684175	6.9176E‐05
	Average values of 3 & 4 Tracer	0.91	0.06	0.25	96.53
	Average values of 3 & 4 *T. gondii*	1.62	0.04	0.15	77.77
SL – Lewiston No Surfactant	BTCs of Columns 5 and 6				
	5 Tracer	0.75	0.057684884	0.240176776	119.0863153
	5 *T. gondii*	0.05	0.01681817	0.129684886	16.61089665
	6 Tracer	0.83	0.093039833	0.305024316	92.61818376
	6 *T. gondii*	4.04	0.093527565	0.305822767	0.115845379
	Average values of 5 & 6 Tracer	0.79	0.08	0.27	105.85
	Average values of 5 & 6 *T. gondii*	2.05	0.06	0.22	8.36
SL – Lewiston Surfactant	BTCs of Columns 7 and 8				
	7 Tracer	1.32	0.04160665	0.203977082	38.17618286
	7 *T. gondii*	0.31	0.196457474	0.443235235	121.293713
	8 Tracer	0.75	0.011111853	0.105412773	37.47634063
	8 *T. gondii*	3.14	0.119199194	0.345252362	−7.696982937
	Average values of 7 & 8 Tracer	1.04	0.03	0.15	37.83
	Average values of 7 & 8 *T. gondii*	1.73	0.16	0.39	56.80
LS – Gilford No Surfactant	BTCs of Columns 9 and 10				
	9 Tracer	0.68	0.049746758	0.223039813	89.83630445
	9 *T. gondii*	0.01	0.011208668	0.105870997	57.4054684
	10 Tracer	0.77	0.064097947	0.253175724	106.3200108
	10 *T. gondii*	0.12	0.351736238	3.354930644	14.5583119
	Average values of 9 & 10 Tracer	0.73	0.06	0.24	98.08
	Average values of 9 & 10 *T. gondii*	0.06	0.18	1.73	35.98
LS – Gilford Surfactant	BTCs of Columns 11 and 12				
	11 Tracer	0.95	0.116975268	0.342016474	111.4324813
	11 *T. gondii*	6.32	0.259486033	0.509397716	−118.3623865
	12 Tracer	1.47	0.085338798	0.292128051	95.72617597
	12 *T. gondii*	0.39	0.077790341	0.278909199	99.42513643
	Average values of 11 & 12 Tracer	1.21	0.10	0.32	103.58
	Average values of 11 & 12 *T. gondii*	3.35	0.17	0.39	−9.47
SL – Greenson No Surfactant	BTCs of Columns 13 and 14				
	13 Tracer	1.14	0.245662077	0.495643094	158.8601214
	13 *T. gondii*	10.71	0.976902692	0.988383879	−319.5828369
	14 Tracer	0.48	0.231606953	0.4812556	142.4699244
	14 *T. gondii*	0.21	0.022350779	0.14950177	55.2519124
	Average values of 13 & 14 Tracer	0.81	0.24	0.49	150.67
	Average values of 13 & 14 *T. gondii*	5.46	0.50	0.57	−132.17
SL – Greenson Surfactant	BTCs of Columns 15 and 16				
	15 Tracer	1.99	0.014070148	0.118617655	−6.463029262
	15 *T. gondii*	0.48	0.000439061	0.020953783	−0.004951934
	16 Tracer	2.11	0.033051724	0.181801331	10.90335269
	16 *T. gondii*	0.44	0.044235144	0.210321525	50.81880006
	Average values of 15 & 16 Tracer	2.05	0.02	0.15	2.22
	Average values of 15 & 16 *T. gondii*	0.46	0.02	0.12	25.41

Abbreviations: LS, loamy sand; SL, sandy loam.

We finally attempted to predict the predominant fate of the oocysts in the different soils, both with and without surfactant, by calculating the recovery of the parasites in soils and leachates from oocyst concentrations applied in pulses. The predominant fate of oocysts in soils subject to simulated rainfall was their retention in the soil profiles, while the predominant fate of oocysts in soils subject to simulated rainfall with surfactant was their presence in the soil leachates due to transport through the soil. There was however, a great deal of variability in the pulse concentrations, which likely biased several percentages of recovery exceeding the average number of oocysts in pulses, particularly in columns receiving surfactant irrespective of the soil type. Variation in detection of oocysts in both soil and leachate is likely due to the presence of compounds as surfactant and soil organic matter that can interfere with PCR amplification and challenge a more accurate quantification of oocysts in soils (Dumètre & Dardé, 2003). The predominant fate of oocysts in leachate in Gilford and Greenson soils characterized by the presence of surfactant is consistent with enhanced transport in these soils with the addition of surfactant. In Sparta and Lewiston soils, although transport was enhanced by surfactant, the predominant fates of oocysts varied in the surfactant‐containing systems.

Collectively, our results demonstrate the heterogeneous distribution of *T. gondii* oocysts in natural soils, with the highest concentrations typically detected within the 2–4 cm top layers. Considering that one infected cat can excrete millions of oocysts for several days (Dabritz & Conrad, 2010; Dubey, 2001), most of the oocyst concentration lies in the uppermost soil layers. Such ‘hot spots’ of oocysts represent an important source of parasite acquisition by foraging birds and mammals (Gilot‐Fromont et al., 2012). Exposure to soil contaminated with oocysts is one of the main risk factors for infection in people (Cook et al., 2000; Dabritz & Conrad, 2010; Egorov et al., 2018; Jones et al., 2009). Oocysts can however move deeper into the soil columns. Mobilization of oocysts from soils to leachate can lead to contamination of aquatic ecosystems from continental to coastal areas (VanWormer et al., 2013; VanWormer et al., 2014). Although the processes result in the dilution of oocyst concentrations in the receiving waters including drinking water supplies, the low infectious doses (1–10 oocysts) still present a possible infection risk for animals and humans consuming contaminated waters. As demonstrated in our study, surfactants are key factors that considerably enhance the transport of oocysts. Surfactants are present in many household cleaning products and, as a result, may enter soil water using greywater for irrigation (Al‐Jayyousi, 2003; Hamilton et al., 2007). Agricultural practices that would promote the use of greywater for crop irrigation should consider the risk of pathogen dissemination throughout the soil and to receiving waters.

## CONCLUSIONS

Transport and retention of *T. gondii* oocysts have been clearly demonstrated in all four of the soil series examined. These processes varied by soil series and with the inclusion of an anionic surfactant in the system. In our experimental system, the presence of clay particles, organic materials and dissolved ions in the soil‐water solutions and soil matrices likely affected the transport of oocysts. Our study and previous works on *C. parvum* oocysts (Bradford & Bettahar, 2005; Darnault et al., 2003; Darnault et al., 2004; Darnault et al., 2017; Darnault et al., 2022) suggest the critical importance of retention mechanisms in the transport of *T. gondii* oocysts in the subsurface environment. Oocysts could be transported quickly by fingering flow and macropores in unsaturated soils. In situations where rainfall is not intense enough to produce fingering flow or macropores, oocysts could be transported through the soil matrix where they interact with soil by straining and a variety of physiochemical processes. In saturated soils, transport could be controlled by a combination of attachment, detachment and straining. The inclusion of surfactant in our experimental system has induced steric forces on the interactions of soil particles and oocysts, and thus reduced the frequency of oocyst adsorption onto soil particles by coating oocysts and soil particles and reducing air‐water surface tension. Our study highlights the need for additional study of the fate and transport of *T. gondii* oocysts through porous media. Saturated flow experiments for the study of contaminants transport in groundwater resources such as aquifers would allow for prediction of the fate and transport of *T. gondii* oocysts in these environments, and when coupled with soil studies would contribute to improve the public health risk assessment of exposure to pathogens through terrestrial and aquatic systems (Chrysikopoulos et al., 2022).

## AUTHOR CONTRIBUTIONS

**Erin N. Kinsey:** Conceptualization (equal); data curation (equal); formal analysis (equal); investigation (equal); methodology (equal); writing – original draft (equal); writing – review and editing (equal). **Caroline Korte:** Formal analysis (equal); investigation (equal). **Sohib Gouasmia:** Formal analysis (equal); investigation (equal). **Coralie L'Ollivier:** Data curation (equal); formal analysis (equal); funding acquisition (equal); investigation (equal); methodology (equal). **Jitender P. Dubey:** Formal analysis (equal); investigation (equal); methodology (equal); writing – review and editing (equal). **Aurélien Dumètre:** Conceptualization (equal); data curation (equal); formal analysis (equal); funding acquisition (equal); investigation (equal); methodology (equal); writing – review and editing (equal). **Christophe J.G. Darnault:** Conceptualization (lead); data curation (lead); formal analysis (lead); funding acquisition (lead); investigation (lead); methodology (lead); writing – review and editing (lead).

## CONFLICT OF INTEREST STATEMENT

The authors declare that they have no known competing financial interests or personal relationships that could have appeared to influence the work reported in this paper.

## Data Availability

The data that support the findings of this study are available from the corresponding author, Christophe J.G. Darnault, upon reasonable request.
